# The sialotranscriptome of *Amblyomma triste*, *Amblyomma parvum* and *Amblyomma cajennense* ticks, uncovered by 454-based RNA-seq

**DOI:** 10.1186/1756-3305-7-430

**Published:** 2014-09-08

**Authors:** Gustavo Rocha Garcia, Luiz Gustavo Gardinassi, José Marcos Ribeiro, Elen Anatriello, Beatriz Rossetti Ferreira, Higo Nasser Santanna Moreira, Cláudio Mafra, Maria Marlene Martins, Matias Pablo Juan Szabó, Isabel Kinney Ferreira de Miranda-Santos, Sandra Regina Maruyama

**Affiliations:** Department of Biochemistry and Immunology, Ribeirão Preto School of Medicine, University of São Paulo, Ribeirão Preto, SP Brazil; Laboratory of Malaria and Vector Research, National Institute of Allergy and Infectious Diseases, National Institutes of Health, Rockville, MD USA; Department of Maternal and Child and Public Health Nursing, Ribeirão Preto School of Nursing, University of São Paulo, Ribeirão Preto, SP Brazil; Department of Biochemistry and Molecular Biology, Federal University of Viçosa, Viçosa, MG Brazil; School of Veterinary Medicine, Federal University of Uberlândia, Uberlândia, MG Brazil

**Keywords:** Ticks, Amblyomma cajennense, Amblyomma parvum, Amblyomma triste, Salivary gland, Saliva, Transcriptome, RNA-seq

## Abstract

**Background:**

Tick salivary constituents antagonize inflammatory, immune and hemostatic host responses, favoring tick blood feeding and the establishment of tick-borne pathogens in hosts during hematophagy. *Amblyomma triste*, *A. cajennense* and *A. parvum* ticks are very important in veterinary and human health because they are vectors of the etiological agents for several diseases. Insights into the tick salivary components involved in blood feeding are essential to understanding vector-pathogen-host interactions, and transcriptional profiling of salivary glands is a powerful tool to do so. Here, we functionally annotated the sialotranscriptomes of these three *Amblyomma* species, which allowed comparisons between these and other hematophagous arthropod species.

**Methods:**

mRNA from the salivary glands of *A. triste, A. cajennense* and *A. parvum* ticks fed on different host species were pyrosequenced on a 454-Roche platform to generate four *A. triste* (nymphs fed on guinea pigs and females fed on dogs) libraries, one *A. cajennense* (females fed on rabbits) library and one was *A. parvum* (females fed on dogs) library. Bioinformatic analyses used *in-house* programs with a customized pipeline employing standard assembly and alignment algorithms, protein databases and protein servers.

**Results:**

Each library yielded an average of 100,000 reads, which were assembled to obtain contigs of coding sequences (CDSs). The sialotranscriptome analyses of *A. triste, A. cajennense* and *A. parvum* ticks produced 11,240, 4,604 and 3,796 CDSs, respectively. These CDSs were classified into over 100 distinct protein families with a wide range of putative functions involved in physiological and blood feeding processes and were catalogued in annotated, hyperlinked spreadsheets. We highlighted the putative transcripts encoding saliva components with critical roles during parasitism, such as anticoagulants, immunosuppressants and anti-inflammatory molecules. The salivary content underwent changes in the abundance and repertoire of many transcripts, which depended on the tick and host species.

**Conclusions:**

The annotated sialotranscriptomes described herein richly expand the biological knowledge of these three *Amblyomma* species. These comprehensive databases will be useful for the characterization of salivary proteins and can be applied to control ticks and tick-borne diseases.

**Electronic supplementary material:**

The online version of this article (doi:10.1186/1756-3305-7-430) contains supplementary material, which is available to authorized users.

## Background

Arthropods have developed several feeding habits during the course of evolution, and within this taxa, hematophagy evolved independently over millions of years, resulting in significant differences among hematophagous arthropods [1, 2]. Most tick species are obligatory blood-feeding ectoparasites and transmit more infectious agents—bacteria, protozoa and viruses—to humans and animals than any other arthropod vectors [3].

Among the hematophagous ticks, there are 130 species of hard ticks of the genus *Amblyomma* (Acari: Ixodidae) [4], which has considerable medical and veterinary importance in the Americas and the Caribbean [5]. The *A. cajennense* tick is associated mainly with savannah biomes under natural conditions [6, 7] and causes severe infestations in equines. Due to its low parasitic specificity, it also infests cattle, dogs, birds, capybaras [8–10] and humans [11] in the urban and peri-urban areas of Brazil, and it is the vector for *Rickettsia rickettsii,* the causative agent of spotted fever in South America [12]. Recent mitochondrial and nuclear DNA analyses suggest that *A. cajennense* is a complex of six species, such that *A. sculptum* is synonymous with *A. cajennense* ticks found in the coastal and central-western regions of Brazil [13, 14].

The *A. parvum* tick may harbor *Rickettsia* species of yet unknown pathogenicity [15, 16], and it is a potential vector for emerging pathogens, such as *Ehrlichia chaffeensis* (the infectious agent of human monocytotropic ehrlichiosis) [17] and *Coxiella burnetii*, which causes Q fever and chronic endocarditis in humans [18]. This tick species is found almost exclusively in dry areas [19]. It feeds on medium-size rodents, such as the yellow toothed cavy (*Galea musteloideds*) [20], but it is also considered a host generalist, as it feeds on domestic animals including cattle and goats [16].

In contrast to the above-mentioned *Amblyomma* species, the *A. triste* tick is mostly associated with marshes and environments prone to flooding [21–23]. Although the deer *Blastocerus dichotomous* is the main host for *A. triste* adults, domestic animals, wild carnivores and humans may also host *A. triste* (reviewed by Nava *et al.*[24]). Epidemio-ecological studies performed in Argentina, Brazil and Uruguay have reported specimens of *A. triste* infected with *Rickettsia parkeri*[25–28], which raises an important health issue due to suspected human cases of rickettsiosis caused by *R. parkeri*[29].

Successful blood meals and enhanced pathogen transmission result from morphological and biochemical adaptations of the tick salivary glands, which secrete bioactive molecules with profound impacts on host hemostasis and inflammation [30, 31]. Additionally, salivary glands have osmoregulatory functions that enable ticks to survive in different environments for long periods away from the host [32]. Tick saliva has anti-clotting, anti-platelet, vasodilatory, anti-inflammatory and immunomodulatory components [33]. Thus, the components of tick saliva are relevant for global public health, as they may contain novel pharmacologically active compounds and provide possible targets for vaccines against ticks and the diseases they transmit [34].

The characterization of gene expression in tissues (transcriptomes) is a robust strategy to uncover tick salivary gland components and functional mechanisms. Salivary gland transcriptomes, or sialotranscriptomes, of several tick species have been described through the analysis of Expressed Sequence Tags (ESTs) obtained by Sanger sequencing of cDNA libraries [35–41], which generally yield a few thousand ESTs. Next generation sequencing (NGS) technologies allow deeper and more accurate gene expression studies because in contrast to previous sequencing methods, millions of short sequences are produced yielding data at the gigabase rather than kilobase scale of traditional cDNA libraries based on cloning [42]. The NGS transcriptional profiling approach is called RNA-seq. To date, two tick sialotranscriptomes have been described by NGS technologies, resulting in a very detailed catalogue of salivary gland transcripts from *A. maculatum*[43], the gulf coast tick, and from *Ixodes ricinus*, the castor bean tick [44].

Here, we describe the sialotranscriptome of three species of *Amblyomma* ticks, *A. triste*, *A. parvum* and *A. cajennense*, using 454-based RNA-seq. These species present distinct biology in terms of preferred habitats and hosts. The repertoire of tick saliva may be complementary to its preferred host’s homeostatic and immune strategies. Ticks can acquire host genes through horizontal gene transfer [45]; therefore, ticks with different feeding habits may have different repertoires of salivary proteins. A total of six RNA-seq libraries were generated from the salivary glands of nymph and female ticks. For the first time, the transcriptional profile of a tissue from *A. parvum* and *A. triste* ticks has been generated and analyzed. Furthermore, we examined the salivary gland transcripts of a third tick, *A. cajennense*, through the analysis of 67,677 reads, which clustered into 4,604 putative coding sequence (CDS) contigs. Comparison of the six annotated sialotranscriptomes revealed common and unique features in the ticks. The transcript catalogues described herein provide insights to the biology of these tick species, which are important ectoparasites, pathogen reservoirs and vectors for diseases.

## Methods

### Ticks, hosts and sample preparation

The following ticks and hosts were used in this study: *A. parvum:* semi-engorged females (54 ticks) fed on dogs; *A. cajennense:* semi-engorged females (45 ticks) fed on rabbits. One pool of salivary glands (SGs) was obtained for both *A. parvum* and *A. cajennense*. As *A. triste* ticks were evaluated in four parasitological conditions, SGs samples generated four pools designed NGP1 (47 nymphs fed on guinea pigs during first infestation) and NGP2 (90 nymphs fed on guinea pigs during second infestation) to designate the semi-engorged nymph samples; FD1 (87 females fed on dogs during first infestation) and FD2 (47 females fed on dogs during second infestation) to designate the semi-engorged female samples. All tick infestations were performed under laboratory conditions as described previously [16, 46, 47]. Nymphs of *A. triste* and females of *A. cajennense* were removed from their hosts between the third and fourth day post-feeding, and *A. triste* and *A. parvum* females were removed between the fourth and fifth day post-feeding. Within the first hour of collection, ticks were washed and dissected to obtain the SGs under sterile conditions. The SG samples were organized in pools according to the tick species, which afterwards corresponded to the number of libraries sequenced. The pairs of SGs were gently washed in ice-cold phosphate buffered saline (PBS) and stored immediately in RNAlater solution (Ambion Inc., Austin, TX, USA) at 4°C for 24 hours and then at -70°C until RNA isolation.

### RNA isolation

Total RNA was isolated from the six pools of tick salivary glands with a protocol optimized in our laboratory. Briefly, after centrifugation, the SG samples were removed from RNAlater solution, and Trizol reagent (Invitrogen, Life Technologies, CA, USA) was added to the samples, which were then thoroughly blended with an overhead stirrer at high speed. Chloroform was gently mixed with the homogenate, and after centrifugation at maximum speed at 4°C, the resuspended aqueous phase was placed in a clean tube containing 96-100% ethanol and mixed. RNA was purified from the mixture with an SV Total RNA Isolation System Kit (Promega Corporation, Madison, WI, USA) according to the manufacturer's instructions. RNA was quantified using a NanoDrop 1000 Spectrophotometer (Thermo Scientific, Wilmington, DE, USA), and the quality was checked with a 2100 Bioanalyzer (Agilent Technologies Inc., Santa Clara, CA, USA). RNA was stored at -70°C.

### Library preparation and pyrosequencing

The raw sequencing data were generated on the 454-Roche platform by the High-Throughput Sequencing and Genotyping Unit, DNA services branch, Roy J. Carver Biotechnology Center, University of Illinois in Urbana-Champaign, IL, USA. Briefly, messenger RNA was isolated from 20 μg of total RNA from each pool of SGs using an Oligotex mRNA kit (Qiagen, Valencia, CA, USA). The mRNA-enriched fraction was converted to a primary cDNA library with barcoded adaptors compatible with the 454-Roche system. The cDNA libraries were quantified using a Qubit fluorometer (Invitrogen, Life Technologies, CA, USA), and the average fragment sizes were determined using a DNA 7500 chip on the 2100 Bioanalyzer. The adaptor-ligated cDNA libraries were pooled in equimolar concentrations, diluted to 1x10^6^ molecules/μl, subjected to emulsion-based clonal amplification and pyrosequenced on a full plate using the 454 Genome Sequencer FLX system (454 Life Sciences, Roche, Branford, CT, USA) according to the manufacturer’s instructions. Signal processing and base calling were performed using the bundled 454 Data Analysis Software version 2.3.

### Bioinformatic analysis

The raw data were downloaded from the High-Throughput Sequencing and Genotyping Unit website, and the bioinformatics workflow for the pyrosequencing data analysis was performed as previously described [43]. The software was written and provided by JMCR in Visual Basic 6.0 (Microsoft, Redmond, Washington, USA). For functional annotation, the CDS contigs were queried against several databases with the blastx, blastn and rpsblast algorithms [48]. We used the followed databases: 1) the non-redundant protein database (NR) of the National Center for Biotechnology Information (NCBI); 2) Gene Ontology (GO) FASTA subset [49]; 3) Swissprot [50]; 4) tick salivary sequences described previously [33]; 5) custom databases from GenBank containing mitochondrial and rRNA nucleotide sequences; 6) CDD from NCBI [51] containing the SMART [52], KOG [53] and PFAM [54] motifs. The functionally annotated catalog for each sialotranscriptome was manually curated and plotted in a hyperlinked Excel spreadsheet. The three spreadsheets were designated Additional file 1 AF1 (*A. triste*), Additional file 2 AF2 (*A. parvum*) and Additional file 3 AF3 (*A. cajennense*) and are available for download at http://exon.niaid.nih.gov/transcriptome/sm/AF1-Atris-web.xlsx, http://exon.niaid.nih.gov/transcriptome/sm/AF2-Aparv-web.xlsx and http://exon.niaid.nih.gov/transcriptome/sm/AF3-Acaj-web.xlsx, respectively (the weblinks are best visualized with the following browsers: Google Chrome, Mozilla Firefox and Internet Explorer). The calculation for differential transcript abundance between the libraries of the three tick species and for the libraries of *A. triste* ticks under different parasitological conditions (Additional file 1 AF1, columns AH-AQ) was performed with a chi-square (Χ^*2*^) test using the number of reads in each CDS from different libraries; results were considered statistically significant if *P* < 0.05. The raw sequencing files were manipulated with the ShortRead package in Bioconductor software [55].

Hierarchical clustering (HCL) to ascertain the gene expression pattern was performed with the standard statistical algorithm described previously [56], using the percentage of reads for each sialotranscriptome, and represented by the log_2_ transformation of reads + 1. HCL employed Euclidean distance for metric calculations and the average linkage method to indicate the cluster-to-cluster distance in the hierarchical trees, which were displayed as heatmaps. These analyses were performed with MeV (Multi Experiment Viewer) software [57]. To compare the different datasets from *A. cajennense* ticks, one library of SGs (LIBEST_019946 [58]) was downloaded from NCBI dbEST database [59, 60] and a second library (LIBEST_USP-RP) described herein (Additional file 4 AF4) were used for sequence similarity searches against the 454-Roche dataset with the blastn algorithm. The translated amino acid sequences of some ESTs from the first two datasets were obtained with NCBI ORF Finder [61].

Multiple sequence alignments were performed with the Clustal W algorithm [62] using Bioedit software [63]. Phylogenetic analyses were determined using the Neighbor-Joining (NJ) method in MEGA 5.0 software [64], and sequences from other tick species were downloaded from the NCBI database (listed in Additional file 5 AF5). The node support of each clade was evaluated using a bootstrap analysis (1000 replicates), and the evolutionary distances were derived using the Jones-Taylor-Thornton (JTT) method [65].

### Accession numbers

Sequence deposition for *A. triste* [GenBank: GBBM01000001 - GBBM01008098 (BioProject 241269)], for *A. parvum* [GenBank: GBBL01000001 - GBBL01002838 (BioProject 241271)] and *A. cajennense* [GenBank: GBBK01000001 - GBBK01005770 (BioProject 241272); JZ718403 – JZ718940].

### Ethical approval

The experiments were approved by Animal Experimentation Ethics Committee of the Federal University of Uberlândia, protocol #033/14, according to Ethical Principles on Animal Research of the National Council for the Control of Animal Experimentation (CONCEA).

## Results and discussion

### Overview of the annotated sialotranscriptomes of *A. triste, A. parvum* and *A. cajennense* ticks

We generated and analyzed the transcriptional profile of salivary glands (SG) from three neotropical hard ticks belonging to the *Amblyomma* genus, obtained from six 454-based RNA-seq libraries: one library from *A. parvum* female ticks fed on dogs, one library from *A. cajennense* female ticks fed on rabbits and four libraries from *A. triste* ticks. The sialotranscriptome of the latter species addressed different parasitological conditions including the developmental stage of the tick, the type of host that each stage prefers [23] and the status of host exposure to ticks and anti-tick immunity. This transcriptome was thus constructed with SGs from nymphs fed on naïve guinea pigs (NGP1), nymphs fed on guinea pigs undergoing a second tick infestation (NGP2), females fed on naïve dogs (FD1) and females fed on dogs undergoing a second infestation (FD2).

The *A. parvum* library yielded 243,567 pyrosequencing reads, and the *A. cajennense* yielded library 67,677 pyrosequencing reads. The four combined *A. triste* libraries yielded 1,195,924 pyrosequencing reads (data not shown). Only the pyrosequencing reads longer than 149 nucleotides were used to assemble primary contigs. The primary assembled contigs (minimum of 3–4 reads per contig) were analyzed to extract putative CDS, and the CDS contigs were used to annotate each sialotranscriptome. The CDS contigs, categorized according to their putative function, were plotted in hyperlinked Excel spreadsheets available as additional files for download: *A. triste* (Additional file 1: AF1), *A. parvum* (Additional file 2: AF2) and *A. cajennense* (Additional file 3: AF3). The annotation of the CDS contigs were performed automatically using a suite of customized programs written in Visual Basic and afterwards manually curated and refined. The functional annotation relied on significant similarities (determined by BLAST algorithms, considering only high identity scores and low E-values) between CDS contigs and sequences in public protein databases and customized databases of known and predicted tick proteins.

The *A. triste* sialotranscriptome was generated from four libraries and consisted of 7,532 CDS contigs (90,367 reads) for NGP1, 7,391 CDS contigs (85,871 reads) for NGP2, 9,311 CDS contigs (120,717 reads) for FD1 and 9,004 CDS contigs (145,801 reads) for FD2. Together, *A. triste* libraries produced a total of 11,240 CDS from 442,756 reads (Table 1 and Additional file 1: AF1). The *A. parvum* sialotranscriptome contained 3,796 CDS contigs assembled from 104,817 reads (Table 1 and Additional file 2: AF2). The *A. cajennense* sialotranscriptome contained 4,604 CDS contigs assembled from 67,677 reads (Table 1 and Additional file 3: AF3).

**Table 1:** **General distribution of reads in coding sequence contigs for the sialotranscriptomes of three** ***Amblyomma*** **ticks**

	***Amblyomma cajennense***				***Amblyomma parvum***				***Amblyomma triste*** *			
Category	CDS^a^	Reads^a^	Reads/CDS	Reads^b^	CDS^a^	Reads^a^	Reads/CDS	Reads^b^	CDS^a^	Reads^a^	Reads/CDS	Reads^b^
Secreted	1,015	16,480	16.2	24.4	493	22,796	46.2	21.7	1,861	76,193	40.9	17.2
Housekeeping	2,805	44,246	15.8	65.4	2,653	65,952	24.9	62.9	6,854	305,129	44.5	68.9
Unknown	338	3,245	9.6	4.8	272	10,739	39.5	10.2	1,071	26,373	24.6	6.0
Unknown secreted	424	3,509	8.3	5.2	359	5,141	14.3	4.9	1,345	33,418	24.8	7.5
Transposable Elements	16	38	2.4	0.1	19	189	9.9	0.2	97	1,405	14.5	0.3
Viral	6	159	26.5	0.2	-	-	-	-	10	193	19.3	0.04
*Leishmania*-related	-	-	-	-	-	-	-	-	2	45	22.5	0.01
**Total**	4,604	67,677			3,796	104,817			11,240	442,756		

a. total number; b. percentage; *Combined data from the four libraries of *A. triste*: salivary glands from nymph ticks fed on guinea pig during first (NGP1) and second (NGP2) infestation; salivary glands from female ticks fed on dogs during first infestation (FD1) and second (FD2) infestation.

Transcripts in the three sialotranscriptomes were classified into seven main categories: a) *Secreted*, having a signal peptide and/or significant BLAST hits with secreted proteins; b) *Housekeeping*, constitutive genes required for the basal maintenance of tissues; c) *Unknown* and d) *Unknown Secreted*, which had non-significant BLAST hits or no matches against any database, being that the latter category (d) contained signal peptide sequences; e) *Transposable Elements*, mobile elements in genomes; f) *Viral*, transcripts with similarities to viral proteins and g) *Leishmania-related*, transcripts with similarities to *Leishmania* proteins. These data are numerically summarized in Table 1.

On average, 20% of the reads in the three sialotranscriptomes were annotated as putative secreted proteins, whereas the majority of the reads (ranging from 62.9% to 68.9%) were annotated as housekeeping genes (Table 1). The *Unknown* and *Unknown Secreted* categories together presented an average of 13% of reads, and this relatively low proportion is a consequence of previous efforts to describe transcripts in salivary glands from several tick species [33].

Because *Amblyomma* ticks are vectors of several microorganisms that are important public health threats, we examined the sialotranscriptomes for CDS related to microbes. The *A. triste* and *A. cajennense* sialotranscriptomes contained 10 and 6 CDSs, respectively, that matched viral-like proteins (Additional file 1: AF1 and Additional files 3: AF3). Forty-five reads were annotated into two CDS coding for *Leishmania*-like proteins in the *A. triste* sialotranscriptome (Additional file 1: AF1). Interestingly, several reports describe the presence of *Leishmania* parasites in ticks [66]. Additionally, we recovered a CDS from *A. parvum* (Ap-19721) that was primarily annotated as ferredoxin protein and that best matched *Rickettsia sibirica* (Additional file 2: AF2), an etiologic agent of North Asian tick typhus. Rickettsial strains phylogenetically related to *R. parkeri*, *R. africae*, and *R. sibirica* have been found in several *Amblyomma* ticks in Brazil [67]. Collectively, these findings support the role of *Amblyomma* ticks as pathogen vectors.

In total, eighty-seven protein families (602,347 reads) were shared within all *Amblyomma* libraries (Additional file 1: AF1, Additional file 2: AF2 and Additional file 3: AF3). Some protein families that were absent in a given sialotranscriptome may be expressed in different developmental stages or even are supplied by members of other families that are functionally redundant.

### Comparative analysis of the *A. triste*, A*. parvum* and *A. cajennense* sialotranscriptomes

**Figure 1:**
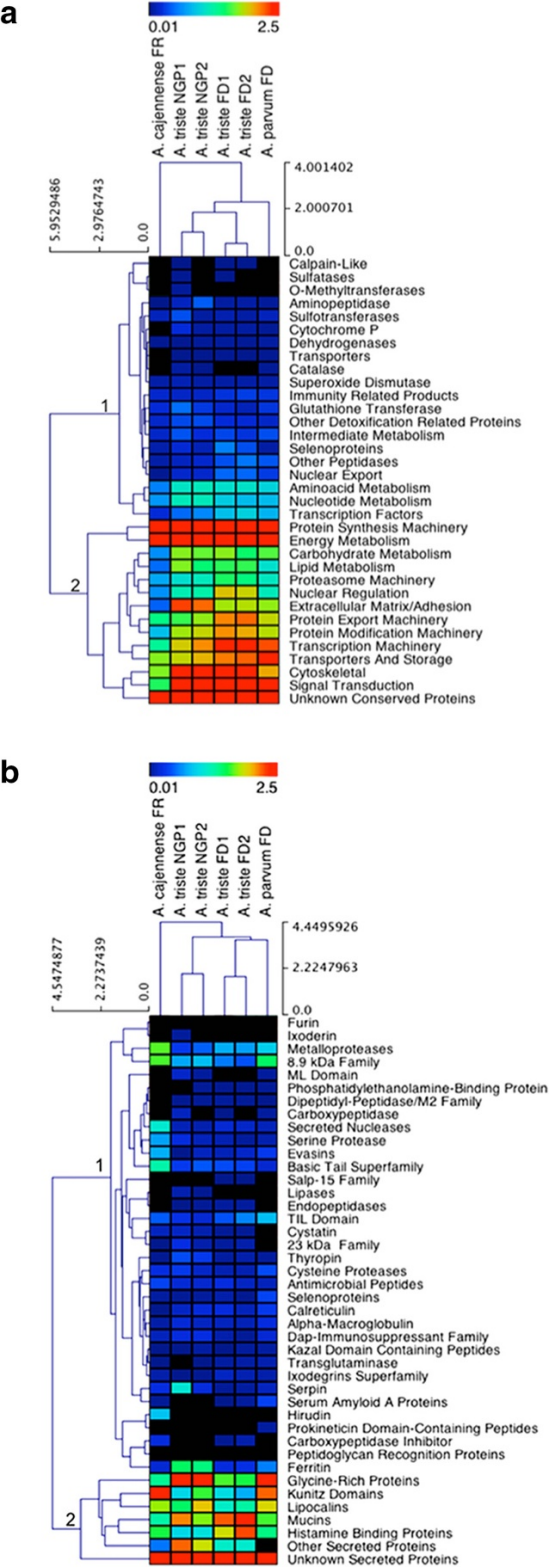
**Hierarchical clustering of putative protein families from the sialotranscriptome of** ***Amblyomma*** **ticks.** The heatmaps display the expression level of the protein families (rows) for each sialotranscriptome (columns). **a)** Families classified as housekeeping proteins. **b)** Families classified as secreted proteins. The color scale represents the log_2_ transformation of % of reads + 1. The transition of colors from blue to red represents an increase in the % of reads. Black color indicates the % of reads <0.01; red color indicates the % of reads ≥ 4.5. The length of branches in hierarchical trees indicates the degree of similarity between objects, either tick libraries (columns) or protein families (rows). Side scales represent the node height (cluster-to-cluster distance). NGP1: nymphs fed on tick-naïve guinea pigs; NGP2: nymphs fed on guinea pigs undergoing a second infestation; FD1: females fed on tick-naïve dogs (first infestation) and FD2: females fed on dogs undergoing a second infestation. FR: females fed on rabbits. The clustering method used Euclidian distance with average linkage.

The *Secreted* and *Housekeeping* categories contained several functional subcategories, and most of these were common between two or three sialotranscriptomes. To understand how the transcriptional profile of these different functional subcategories changes across the three *Amblyomma* species, we performed a hierarchical clustering using the abundance of transcripts (relative percentage of reads) for each sialotranscriptome. For the *A. triste* sialotranscriptome, we deconstructed it according to the experimental conditions (two developmental stages on once- and twice-infested hosts).The expression data are displayed as heatmaps where the black to red colors indicate low and high expression levels, respectively (Figure 1). The abundances of the 33 subcategories of housekeeping genes together with the Unknown category genes were clustered in two main groups according to the expression level (Figure 1a, transcript clusters numbered 1 and 2). For instance, weakly expressed transcripts encoding calpain-like proteins, sulfatases, O-methyltransferases and catalases are in the first cluster, whereas the second cluster is composed of more abundant transcripts such as those encoding protein synthesis machinery, energy metabolism, transcription machinery, cytoskeletal and signal transduction proteins.

Regarding the clustering of *Amblyomma* species using the expression patterns of housekeeping genes (Figure 1a, library clustering from right to left), the libraries from *A. parvum* and *A. triste* formed the main cluster, where the two minor clusters were arranged according to the host and life stage of *A. triste* ticks, nymphs fed on guinea pigs (NGP) or females fed on dogs (FD) (Figure 1a, library clustering from right to left). Notably, libraries from different tick species using the same host for blood meals were clustered closer (i.e., more similar), as observed for *A. parvum* FD and *A. triste* FD1 and FD2 libraries (Figure 1a). Additionally, the branch length of the sub-cluster formed with *A. triste* FD libraries indicated that the expression pattern was slightly more similar for female ticks than nymph ticks. Although the patterns in the four *A. triste* libraries were similar, the FD libraries were enriched for transcription machinery, protein export machineries and transport and storage, while the NGP libraries had a higher abundance of extracellular matrix/adhesion-related transcripts. The *A. cajennense* library was externally positioned and distant from the other female tick libraries. In addition to the species difference, this distance (i.e., less similarity with the others) might have been influenced by the blood meal source for this tick, which was fed on rabbits (FR). Almost all *A. cajennense* transcript families had lower expression compared with the other libraries. These transcription profiles suggest that the amount and content of proteins produced by salivary glands depends on the tick species and life stage as well as the host background.

Although the *Secreted* category contained about one-fifth of the transcripts (Table 1), many diverse protein families were observed, and approximately double the number of housekeeping protein families were present, considering all subcategories of putative secreted proteins (Additional file 1: AF1, Additional file 2: AF2 and Additional file 3: AF3). Despite the lower numbers of transcripts than the housekeeping genes, this large repertoire with relatively small quantities of secreted proteins may have driven the performance of ticks during blood meals. Thus, tick saliva relies on the specificity of this secreted protein repertoire, which in turn may vary between tick species, which live in different habitats and have a range of preferred hosts.

The hierarchical clustering of the putative secreted transcripts resulted in distribution pattern similar to housekeeping protein families, such that *A. cajennense* was placed as external member and the *A. triste* FD libraries were grouped with the *A. parvum* FD library instead of the *A. triste* NGP libraries (Figure 1b, library clustering)*.* In addition, the branches were longer between the *A. triste* libraries in the hierarchical tree for secreted transcripts, reflecting less similarity. In general, the expression profile of transcripts encoding putative secreted proteins was less well defined across species than the housekeeping genes. The transcription of secreted proteins may be more prone to parasitological changes (e.g., nymphs versus females) as well as the different host environments (e.g., dogs or guinea pigs, once- or twice-infested).

Compared with the housekeeping transcripts, the expression level of genes encoding putative secreted proteins was lower. Most transcripts encoding secreted proteins were expressed at a low level (Figure 1b, transcript cluster 1), whereas a few families showed medium to high expression levels (Figure 1b, transcript cluster 2). Additionally, HCL analysis showed that some cluster 1 transcripts encoding secreted proteins differed significantly between the *A. cajennense* and the *A. parvum* and *A. triste* libraries (Table 2).

**Table 2:** **Transcripts coding secreted proteins differentially represented between** ***A. cajennense*** **,** ***A. parvum*** **and** ***A. triste*** **ticks**

Protein families	Number of reads											
	***A. cajennense***	***vs***		***A. parvum***	***A. cajennense***	***vs***		***A. triste*** ^#^	***A. parvum***	***vs***		***A. triste*** ^#^
	Obs.	Exp.	Obs.	Exp.	Obs	Exp.	Obs.	Exp.	Obs.	Exp.	Obs.	Exp.
Metalloproteases	1,439*	946.1	906	1,399	1,439*	835.5	828	1,431	906*	775.1	828	959.9
8.9 kDa Family	1,400*	1,268	1,832	1,964	1,400*	712	582	1,270	1,832*	1,122	582	1,292
ML Domain	0	14.9	38*	23.1	0	3.6	10	6.4	38*	22.3	10	25.7
Cystatin	62*	24.3	0	37.7	62*	33	30	59	0	13.9	30*	16.1
23 kDa Family	8	3.1	0	4.9	8	18,3	43*	32.7	0	20	43*	23
TIL domain	256	414.7	801*	642.3	256	243.6	422	434.4	801*	568.4	422	654.6
Basic tail Superfamily	941*	427.7	149	662.3	941*	481.7	400	859.3	149	255.1	400*	293.9
Carboxypeptidases	0	14.1	36*	21.9	0	5	14*	9	36*	23.2	14	26.8
Cystein Proteases	132	194.5	349*	286.5	132*	81.5	89	139.5	349*	195.5	89	242.5
Serine Proteases	424*	199.3	69	293.6	424*	188.9	88	323.1	69	70	88	86.9
Secreted Nucleases	784*	352.5	87	518.5	784*	354	175	604.9	87	116.9	175*	145
Antimicrobial Peptides	202*	116.2	94	179.8	202*	156	226	271.9	94	145.5	226*	174.5
Evasins	459*	284.1	265	439.9	459*	198.3	93	353.7	265*	166.4	93	191.6
Thyropin	12	18	34	28	12	21.9	49*	39.1	34	38.6	49	44.4
DAP-36 immunosuppressant family	58	82.8	153*	128.2	58*	44.9	67	80.1	153*	102.2	67	117.8
Kazal domain containing peptides	9	20	42*	31	9	10.8	21	19.2	42*	29.3	21	33.7
Phosphatidylethanolamine-binding protein	2	11.8	28*	18.2	2	10.8	28*	19.2	28	26	28	30
Salp-15 family	0	0	0	0	0	6.5	18*	11.5	0	8.4	18*	9.6
Serpin	133*	89.5	95	138.5	133*	60.4	35	107.6	95*	60.4	35	69.6
Serum Amyloid A Proteins	17	127.5	308*	197.5	17	12.2	17	21,8	308*	151	17	174
Hirudin	533*	211.1	5	326.9	533*	194.3	8	346.7	5	6	8	7
Prokineticin domain-containing peptides	0	18.4	47*	28.6	0	1.8	5	3.2	47*	24.2	5	27.8
Carboxypeptidase inhibitor	121*	49	4	76	121*	53.5	28	95.5	4	14.9	28*	17.1
Ferritin	103	245.2	522*	379.8	103	113.9	214	203.1	522*	342.1	214	393.9
Glycine-rich proteins	1,026	2,888	6,336*	4,474	1,026	1,288	2,512*	2,250	6,336*	4,021	2,512	4,827
Mucins	957	1,218	2,147*	1,886	957	1,923	4,396*	3,430	2,147	3,041	4,396*	3,502
Histamine binding proteins	1,311*	1,170	1,671	1,812	1,311	1,626	3,216*	2,901	1,671	2,271	3,216*	2,616
Kunitz Domains	4,033*	3,122	3,748	4,659	4,033*	1,916	1,190	3,307	3,748*	2,210	1,190	2,728
Lipocalins	1,585	1,745	2,864*	2,703	1,585*	1,103	1,486	1,968	2,864*	2,022	1,486	2,328

Calculation was performed with chi-square test (χ^2^) using number of reads assigned for protein families, according functional annotation (Additional file 1: AF1, Additional file 2: AF2 and Additional file 3: AF3); Obs.: Observed; Exp.: Expected. The higher number of reads observed between the comparisons are highlighted in bold if statically significant according the χ^2^ test result (* *P* < 0.05). **#**: The comparisons were performed only with the library of *A. triste* female ticks fed on dog during the first infestation (FD1).

Among the families with low expression (Figure 1b, protein cluster 1), we highlight the metalloproteases, Salp-15, antimicrobial peptides and evasins. The metalloproteases were represented by reprolysin, neprilisin-type, and other proteins that have metal-dependent proteolytic activity to gelatin, fibrinogen and fibronectin, which could help regulate host inflammatory and immune responses [68, 69].

Salp-15 protein was first described in *I. scapularis* saliva as an immunomodulatory protein that inhibits CD4^+^ T cell differentiation through its interaction with the CD4 co-receptor and favors the transmission of diverse *Borrelia* species to their hosts [70, 71]. Salp-15 was only found in FD libraries from *A. triste*. As *A. triste* is a vector of human rickettsiosis [23], the putative Salp-15 might interfere with the host immune response, supporting both the hematophagous ectoparasite as well as the pathogenic bacteria carried by the tick. Hence, it will be interesting to further examine *A. triste* as a pathogen vector.

The superfamily of antimicrobial peptides is formed by CDSs encoding defensins, microplusins, lysozymes and antigen 5 families. Ticks produce antimicrobial peptides as part of their innate immunity; these peptides control and combat invading microorganisms [72–74]. These families were expressed in the sialotranscriptomes of all three *Amblyomma* species, indicating their importance for the protection of the ticks against potentially harmful microorganisms during hematophagy. Notably, these transcripts were differentially expressed, with higher expression in *A. cajennense* followed by the *A. triste* and *A. parvum* female libraries (Table 2).

All libraries contained CDSs encoding evasin, a salivary protein previously identified in *R. sanguineus* ticks [75]. Evasins hinder the host immune response by binding chemokines and impairing cell migration [75]. These transcripts were more highly expressed in the *A. cajennense* and female libraries compared with the *A. triste* nymph libraries (Figure 1b and Table 2).

Aside from unknown secreted proteins, the most abundant transcripts encoding secreted proteins were members of the GRP category (Figure 1b, transcript cluster 2). Significant differences in GRP expression were observed between female libraries of the three species of ticks; *A. cajennense* females (FR) contained significantly less GRP transcripts than *A. parvum* females and *A. triste* fed on dogs (Table 2). *A. triste* NGP libraries also had an abundance of GRP expression (Figure 1b). We previously showed that the amount and types of GRP are related to the biology of the tick species, namely, whether it is monoxenous or heteroxenous and whether it has a short or long hypostome [76]. In this study, all ticks were heteroxenous Longirostrata ticks, and *A. triste* and *A parvum* females were fed on dogs. One possible explanation is the different ecosystems where these ticks live. *A triste* is found on hosts living in marshes, where humidity is high, and *A. parvum* is found in the dry savannah. In addition to their roles in tick attachment to host skin, GRPs are also hygroscopic proteins. The ticks in our experiments were reared in controlled laboratory conditions, but basal expression of GRP may have been maintained each species.

The Kunitz domain family is subclassified according to the number of KU domains in each sequence (i.e., Monolaris, Bilaris, Trilaris, Tetralaris, Pentalaris and Hexalaris). Members of this family are abundant in the sialotranscriptomes of hematophagous arthropods, such as flies and ticks [33, 77, 78], and typically, they are protease inhibitors that impair various processes including blood coagulation and promote vasodilatation [79]. Therefore, this family of proteins facilitates hematophagy and plays an important role during tick engorgement. The Kunitz domain family was represented by significantly fewer transcripts in the *A. triste* FD transcriptome (Table 2). In all developmental stages, hosts and their immune statuses, this family was expressed at a lower level in *A. triste* than in *A. cajennense* and *A. parvum* (Figure 1b and Table 2). This finding merits further investigation to determine if other mediators of antihemostasis may substitute for this family in the sialome of *A. triste*. Another hypothesis is that temporal differences affect the coagulation and fibrinolytic systems of different mammalian hosts [80]: whilst *A. triste* and *A. parvum* females were both fed on dogs, the genetic composition of these species of ticks may be complementary to the hemostatic mechanisms of their preferred natural hosts, ungulates and rodents, respectively.

Serine/threonine-rich proteins containing potential glycosylation sites are termed mucins. Mucins are mainly present in mucosal tissues and function as mechanical barriers to damage from pathogens and toxins [81]. Transcripts encoding members of this protein family have been recovered in several tick sialotranscriptomes [35, 39, 43]. We found that these transcripts were highly abundant in all *A. triste* libraries (Figure 1b). Within the female libraries of *Amblyomma* ticks, these transcripts were significantly higher in the FD libraries from *A. triste* and *A. parvum* (Table 2). Although this evidence suggests that the host may influence the expression of mucins, for instance, *A. cajennense* FR library had fewer of these transcripts than other libraries (Figure 1b and Table 2), it is possible that other protein families may overcome the protection provided by them, such as antimicrobial peptides that were significantly more transcribed in *A. cajennense* (Figure 1b and Table 2).

Transcripts for histamine-binding proteins (HBPs) were more abundant in *A. triste* females fed on dogs (FD1 and FD2), and the expression of other lipocalins was significantly higher in *A. parvum* compared with *A. cajennense* and *A. triste* females (Figure 1b and Table 2). Histamine is an important mediator of inflammation, and its concentration varies depending on the host skin. Tick histamine-binding proteins have a high affinity for histamine [82] and they suppress inflammation caused by tick bites and saliva. HBPs was previously characterized in the saliva of the *R. appendiculatus* tick [83], and transcripts encoding HBP and lipocalin are also present in several other tick sialotranscriptomes [36, 39, 43].

Additionally, several CDS contigs displayed a high level of identity with protein sequences previously described in the sialotranscriptomes of other species in the *Amblyomma* genus, namely, *A. maculatum*[43] and *A. variegatum*[41], which employed the same bioinformatics strategy for the sequence assembly. Most of the characterized salivary proteins for the *Amblyomma* genus—for example, Amblin [84], Variegin [85] and Serpins [86, 87]—have anti-hemostatic functions. One CDS similar to Amblin was present in the *A. triste* sialotranscriptome (At-143156), and we found sequences in the sialotranscriptome of *A. triste* (At-34965, At-35820 and At-30336) and *A. cajennense* (Ac-23315) annotated as serpins with at least 82% of amino acid identity with the serpins/lospins from *A. americanum*[86, 87]. CDSs similar to the well-characterized thrombin inhibitor Variegin could not be found in our analyses, because any small protein sequences (less than 49 amino acids) were excluded during bioinformatics steps; nevertheless, this does not mean that transcripts for Variegin or any other small proteins were not expressed. CDSs similar to proteins that presents non-anti-hemostatic functions, —such as ferritins, iron carriers found in several ticks [88], and Hebraein [72], an antimicrobial described for *A. hebraeum*— were also presented in *A. parvum* (Ap-3247, 98% identical to *A. americanum* ferritin) and *A. cajennense* (Ac-5954, 98% identical to *A. americanum* ferritin and Ac-6284/Ac-6285, 86% identical to Hebraein). Protein sequence similarities with other *Amblyomma* species can be explored in the three annotated sialotranscriptomes (see BLAST results for Acari protein database in the Additional file 1: AF1, Additional file 2: AF2 and Additional file 3: AF3).

### *Amblyomma triste*: changes in the sialotranscriptome depend on host immunity and the life stage of the tick

**Figure 2:**
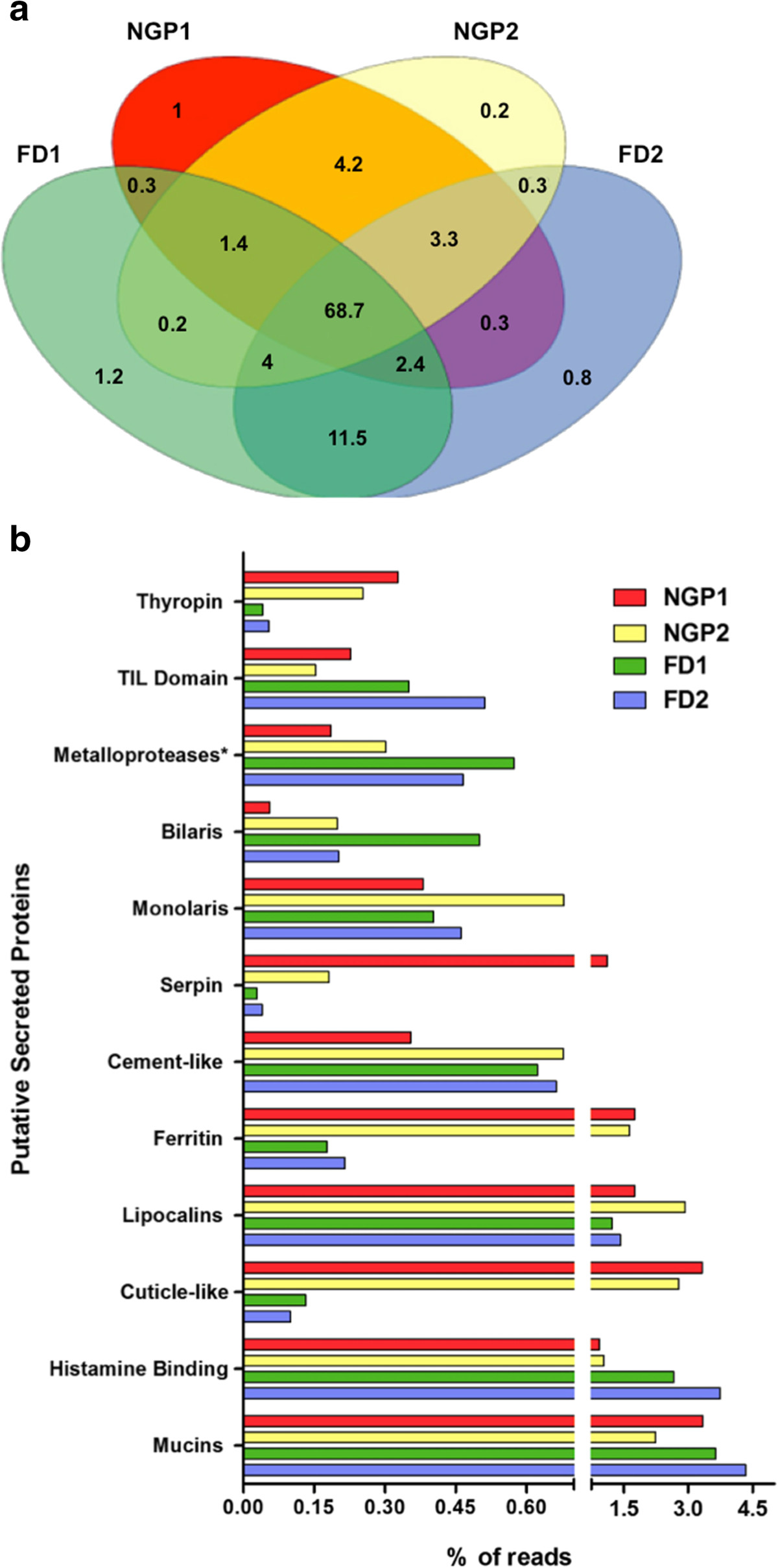
**Comparative analyses of the sialotranscriptome of** ***Amblyomma triste*** **ticks. a)** Venn diagram showing the percentage of common and unique reads in the salivary glands of *A. triste* during blood feeding in four parasitological conditions. NGP1: nymphs fed on naïve guinea pig; NGP2: nymphs fed on guinea pig undergoing a second tick infestation; FD1: females fed on naïve dogs and FD2: females fed on dogs undergoing a second tick infestation. **b)** Abundance of some transcripts coding for putative secreted proteins across the different conditions addressed herein. All of these protein families were differently expressed between libraries, NGP1 *vs.* NGP2 and FD1 *vs.* FD2, according to a chi-square test (*P* < 0.05) using the number of reads for each library in a given functionally annotated contig. *Metalloproteases of the reprolysin family.

The sialotranscriptomes of *A. triste* ticks in four different biological conditions (NGP1, NGP2, FD1 and FD2) were compared to determine which transcripts were shared and unique in these four libraries (Figure 2). Most transcripts (68.7%) were shared by all four libraries. In the libraries from ticks feeding on hosts infested once or twice and thus presenting different levels of immunity, the total number of unique transcripts was decreased in the nymphs (from 1% in NGP1 to 0.2% in NGP2) and adults (from 1.2% in FD1 to 0.8% in FD2). Only 0.3% of the transcripts are uniquely shared between the libraries representing the first (FD1 and NGP1) or second (FD2 and NGP2) tick infestation, regardless of the host background or the tick life stage. A small increase in uniquely shared reads was observed when ticks in the same life stage were feeding on the same host, for example, overlaps between FD1 and FD2 (11.5%) or NGP1 and NGP2 (4.2%) (Figure 2a).

Among the numerous families annotated within the *Secreted* category, we emphasize the abundance of 12 major families that were significantly differentially expressed between the libraries (Figure 2b and Additional file 1: AF1). The CDSs for Thyropin, Serpin, Ferritin and Cuticle-like protein were more abundant in nymphs. Tick serpins are serine protease inhibitors that play key roles in the regulation of blood coagulation and inflammation through the inhibition of cathepsin G, chymase and vertebrate elastase [89, 90]. They were highly expressed in NGP1, with an average 10-fold decrease in NGP2 (Figure 2b). This decrease might be caused by the acquired resistance of guinea pig hosts during the second infestation, which affected the gene expression in NGP2 by unknown mechanisms.

The optimal development of eggs and offspring viability requires the ingestion of a large volume of blood by female ticks. Due to the large amount of blood consumed, hematophagous arthropods must process a high content of iron in hemoglobin, which in excess becomes toxic. In this context, tick ferritins neutralize iron toxicity through the metabolism and storage of iron [91, 92], thus allowing successful parasitism. Ferritin transcripts were significantly more abundant in the sialotranscriptome of the nymphal stage (Additional file 1: AF1, columns AH-AQ), possibly in preparation for the female stage of development in which rapid engorgement with blood and iron accumulation occurs.

Cuticle proteins are also an essential factor for parasite success. In addition to forming part of the external tegument of the tick exoskeleton, the cuticle lines internal tissues, such as salivary gland ducts, trachea and the digestive tract [93]. Thus, cuticle is important for molting, and it supports physiological changes in internal tissues during parasite development and hematophagy.

In the *A. triste* sialotranscriptome, we observed that transcripts encoding secreted ferritins and cuticle-like proteins had the same expression patterns in libraries from the same tick life stage (NGP1 and NGP2; FD1 and FD2). Both transcripts were highly expressed in the nymphal stage, which had 10-to-20-fold more reads than the female libraries (Figure 2b). The potential high expression of these two types of proteins on nymphs fed on guinea pig hosts, either once or twice infested, may explain at molecular level why specific tick life stages have different performances depending on the host species [46]. For instance, *A. triste* nymphs are more efficient parasites when they are fed on guinea pigs, whereas capybaras are best for *A. triste* female ticks.

Conversely, other transcripts were more abundant in the salivary glands of female ticks, such as those encoding metalloproteases, TIL domain-containing proteins and histamine-binding protein (Figure 2b). In *I. scapularis*, metalloproteases in saliva inhibit endothelial cell proliferation, angiogenesis and fibrinogenolysis [68, 94], processes important for wound healing at the tick bite site. In addition, metalloproteases are involved in the metabolism of several peptides that regulate inflammation and immunity [69]. Thus, tick metalloproteases are essential for a complete blood feeding. Another secreted protein family important for the “tick hematophagy cocktail” contains the histamine-binding proteins, which were more abundant in female tick libraries. Because dogs are not the preferred hosts for adult ticks, the increased expression of these two proteins (TIL and HBP) in *A. triste* females might be a mechanism to overcome an impaired blood meal, in which females struggle to feed properly on the dog host. Lastly, the TIL domain-containing proteins were also more abundant in female libraries. These cysteine-rich antimicrobial proteins inhibit serine proteases that are important for bacterial growth [73, 95].

While we cannot confirm that the expression patterns are stage-specific or host-specific because expression data from nymphs fed on dogs and from females fed on guinea pigs is not available, the transcripts highlighted above were consistent in the pairs of libraries (NGP1 and NGP2; FD1 and FD2), suggesting that both the life stage and host may be driving gene expression in salivary glands.

### *Amblyomma cajennense*: the contribution of this new sialotranscriptome

The salivary glands of *A. cajennense* female ticks have been the subject of two other gene expression studies, conducted by Batista et al. ([58], here designated LIBEST_019946) and Anatriello et al. [96], but the most recent study has not made the identified sequences publicly available in databases [96]. In addition to a new sialotranscriptome from *A. cajennense* female ticks fed on rabbits containing over 4,600 coding sequences, we briefly report a reanalysis of the sequences from Anatriello’s [96] work (here designated LIBEST_USP-RP dataset). We then compared these two datasets to reveal how our new sialotranscriptome enriched the pre-existing data.

The LIBEST_USP-RP dataset originated from the salivary glands of *A. cajennense* female ticks fed on horses [76, 96] and contains 1,147 ESTs. The dataset was reanalyzed with a previously described bioinformatic pipeline [36], and the annotated high-quality sequences clustered into 505 contigs with stringent similarity parameters (Additional file 4: AF4). Nearly 22% of the ESTs were annotated as putatively secreted and belonged to protein families consisting of proteases, protease inhibitors and cement-like proteins. However, the function of about half of these ESTs is unknown.

**Figure 3:**
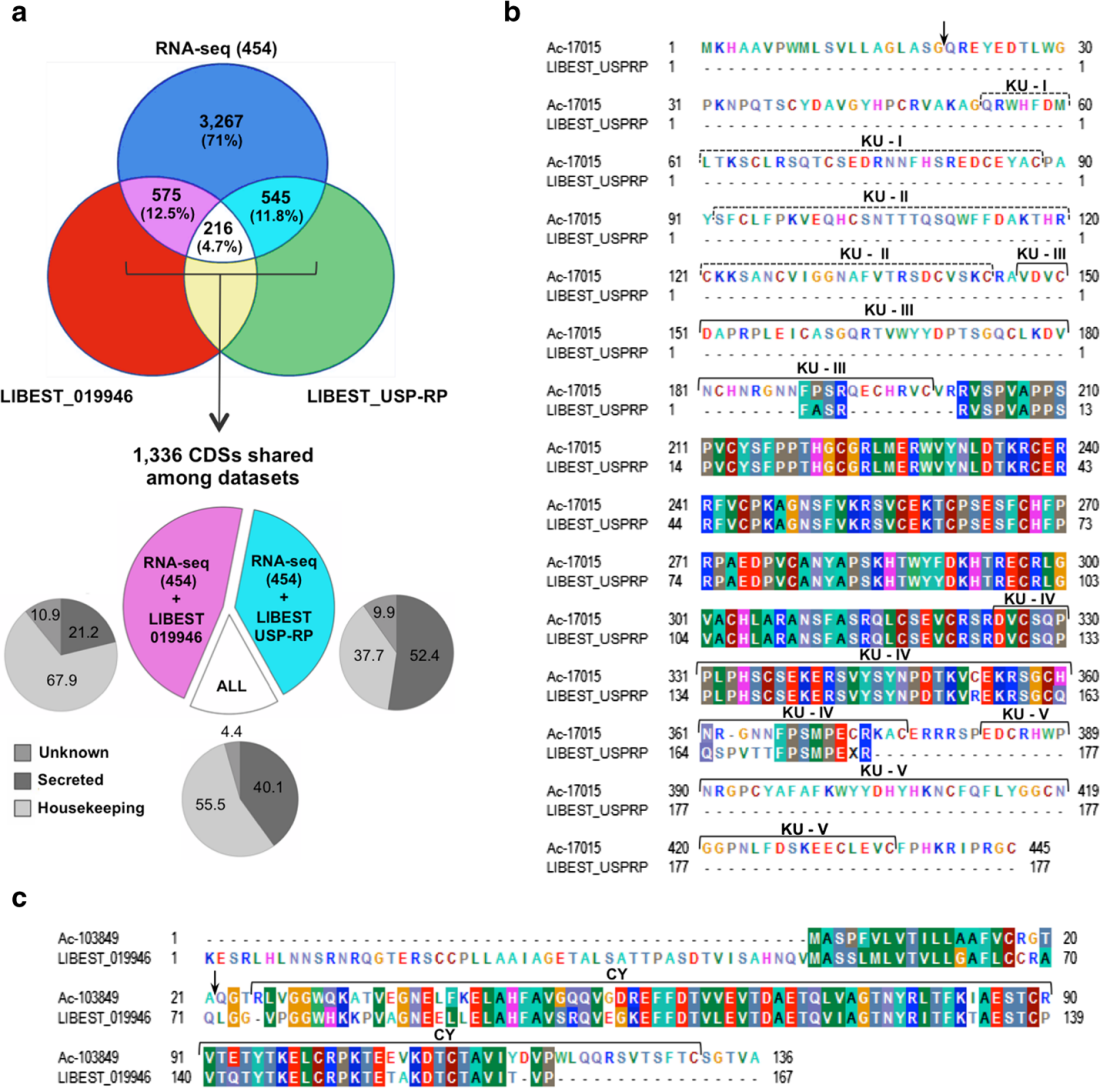
**Comparison between distinct sialotranscriptome of** ***Amblyomma cajennense*** **tick. a)** Venn diagram showing the proportion of CDSs (4,604 annotated in Additional file 3: AF3) identified in the RNA-seq (454) dataset and shared with the LIBEST_019946 (Batista et al., 2008) and LIBEST_USP-RP datasets. The values were based on the number of CDSs with significant matches to the other two datasets using the blastn algorithm. The magenta, cyan and white overlaps represent the sequences shared between the datasets. Approximately 30% of the CDSs (1,336) were contained sequences shared between two or more libraries. The proportion of shared proteins putatively secreted in the saliva, with housekeeping or unknown functions, are displayed in the small gray pie charts at the bottom. **b)** Sequence alignment of CDS Ac-1705 and ACGLSP02_C10 of the LIBEST_USP-RP dataset. This CDS was annotated as a putative Pentalaris protein, a serine protease inhibitor belonging to Kunitz domain superfamily. Horizontal brackets indicate the Kunitz domains (KU, 53 amino acids) along the sequences (I to V), as identified by the SMART protein database (smart00131). Dashed brackets indicate SMART hits with low scores, whereas solid brackets indicate SMART hits with high similarity scores. **c)** Sequence alignment of CDS Ac-103849 and ACAH15A10 of the LIBEST_019946 dataset. This CDS was annotated as a secreted Cystatin, a cysteine protease inhibitors. Horizontal brackets indicate the Cystatin-like domain (CY, 107 amino acids) identified by the SMART protein database (smart00043). In *b* and *c*, upper arrows indicate the peptide cleavage sites predicted by SignalP Server [97]. Side numbers on block alignments indicate the amino acid position in each sequence. The alignments were produced with Clustal-W using a PAM 250 matrix for amino acid coloring in BioEdit software.

The new sialotranscriptome (Additional file 3: AF3, here designated the RNA-seq (454) dataset) contained many more sequences than the LIBEST_019946 and LIBEST_USP-RP datasets. The two LIBEST datasets contain less than one megabase (MB) of content, whereas the RNA-seq (454) dataset produced over 69 MB, yielding approximately 180 times more sequences (Table 3). From the 4,604 CDSs annotated for RNA-seq (454), almost 30% (1,336) were shared with the other two LIBEST datasets (Figure 3a, see magenta, cyan and white overlaps). This ratio of shared sequences was calculated based on blastn searches using LIBEST_019946 and LIBEST_USP-RP as databases, and every match with an E-value less than 10^-10^ was counted. In fact, the ratio of shared sequences may be lower because the counting was redundant, i.e., many CDSs from the new sialotranscriptome matched the same sequences from one or both LIBEST datasets. Because the CDSs were functionally annotated, we ascertained the main functional categories for each shared sequence. Whereas housekeeping proteins were most prevalent (67.9%) in the RNA-seq (454) and LIBEST_019946 overlap, secreted proteins were the majority of sequences (52.4%) shared by the RNA-seq (454) and LIBEST_USPRP overlapping reads (Figure 3a, pie charts at the bottom). Thus, most transcripts shared by the datasets 454-RNA-seq and LIBEST_USPRP potentially compose the saliva of *A. cajennense*.

**Table 3:** **General characteristics of cDNA libraries from salivary glands of** ***Amblyomma cajennense*** **produced in different conditions**

Library	Tick life stage	Host	No. of sequences	Average size of sequence^a^	Number of Contigs	Total yield in MB^b^
LIBEST_019946^c^	Adult fed females	Rabbits	1,769	472	1,234	0.79
LIBEST_USP-RP^d,e^	Adult fed females	Horses	1,147	493	505	0.57
RNA-seq (454)^f^	Adult fed females	Rabbits	180,857	384	4,604	69.4

^a^size in bases; ^b^size in mega (10^6^) bases; ^c^described by Batista *et al*.[58]; ^d^produced by Anatriello *et al*. [96] and described herein; ^e^for details see Additional file 4: AF4. ^f^Shown values were calculated considering the two Roche 454 libraries described herein (for details regarding contigs see Additional file 3: AF3).

In addition to its 70% novel sequences (Additional file 3: AF3), the new sialotranscriptome supplements the existing sequences for this tick species. For example, we performed protein sequence alignments using the translated nucleotide sequences from the three datasets and two important protein families of protease inhibitors. The CDS Ac-17015 was classified as a putative BPTI/Kunitz-type serine protease inhibitor, which contains five *in tandem* Kunitz (KU) domains within its 445 amino acids (aa). A five-KU domain protein, Penthalaris, is a tick anticoagulant found in the salivary glands of the Lyme disease vector, *Ixodes scapularis*[98]. Not only was Ac-17015 very similar to the aligned region of sequence ACGLSP02_C10 (533 nt, 177 aa; 89.2% identity and 91.4% similarity) from the LIBEST_USPRP dataset, but it also complemented the existing database record with the full-length sequence containing both the N- and C-termini of the putative protein (Figure 3B). Although only one KU domain was detected in the incomplete sequence of the serine protease inhibitor in the LIBEST_USPRP dataset, this putative protein could be considered a Pentalaris-type protein because it presented five KU domains.

Another secreted protease inhibitor addressed by sequence alignment was a member of the Cystatins, a single domain protein family of cysteine protease inhibitors in vertebrates, invertebrates and plants that affect a broad range of biological process [99]. Tick cystatins have been observed in the midgut and/or salivary glands and are most likely important during feeding and blood digestion [100]. The CDS Ac-103849 was classified as a secreted cystatin (136 aa) and was very similar to the aligned region of the ACAH15A10 sequence (505 nt, 167 aa; 59.5% identity and 73.5% similarity) from the LIBEST_019946 dataset (Figure 3c). In addition to the putative 5’ untranslated region, the translated nucleotide sequence of ACAH15A10 had a stop codon (not shown in the alignment) at 10 residues before the initial methionine, most likely due to sequencing error. This is an example of how the new *A. cajennense* sialotranscriptome will complete the existing expression data from salivary glands of this tick. However, we must mention that the two LIBEST datasets also contain important unique sequences produced by biological variations at the tick-host interface, such as tick population, host species and time of blood feeding.

### Phylogenetic analyses of salivary coding sequences with potential immunomodulatory functions

**Figure 4:**
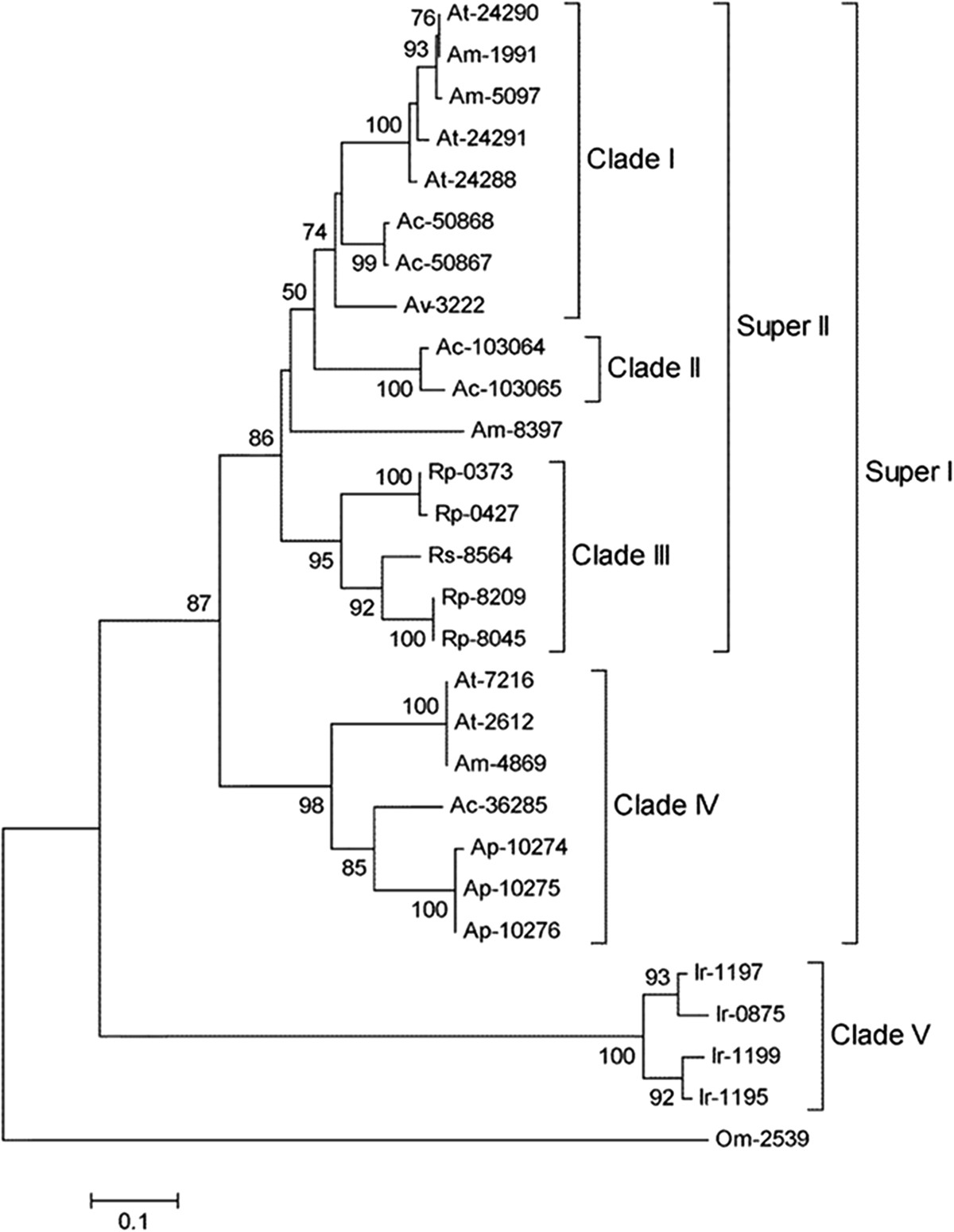
**Phylogenetic analysis of the salivary thyropin proteins from different tick species.** Phylogram resulting from the alignment of 11 full-length protein sequences from the sialotranscriptomes of *A. triste* (At-7216, At-2612, At-24288, At-24290 and At-24291)*, A. parvum* (Ap-10275, Ap-10276 and Ap-10274) and *A. cajennense* (Ac-103065, Ac-50867 and Ac-36285) as well as sequences from other ticks previously deposited in the NCBI protein database, including *A. maculatum* (Am-1991, Am-8397, Am-5097 and Am-4869)*, A. variegatum* (Av-3222)*, Ixodes ricinus* (Ir-1197, Ir-0875, Ir-1199 and Ir-1195), *Rhipicephalus pulchellu* s (Rp-0373, Rp-8209, Rp-0427 and Rp-8045), *R. sanguineus* (Rs-8564) and *Ornithodoros moubata* (Om-2539). The database access numbers used in this analysis are listed in Additional file 5: AF5. The neighbor-joining method with 1,000 bootstrap replicates was used for analysis in MEGA 5.05 software. The numbers on the nodes indicate the bootstrap support. Brackets highlight branches with more than 72% bootstrap support. The bar at bottom indicates 10% amino acid divergence.

The three *Amblyomma* sialotranscriptomes contained the cysteine protease inhibitors called thyropins, which contain thyroglobulin type-1 (Thyr-1) domains and inhibit either cysteine or cation-dependent proteinases [101]. During tick feeding, antigens are exposed to hosts and enter the endocytic pathway of antigen presenting cells (APC), where they are processed and loaded onto the major histocompatibility complex (MHC) class II. Proper folding and trafficking of MHCII is associated with the invariant chain (Ii) molecule, whose Thyr-1 domain-containing isoforms regulate endosomal cathepsins, cysteine proteases implicated in antigen processing [102]. The significant homology between Ii Thyr-1 domains and thyropins suggests that this protein family controls host immune factors. The *A. triste*, *A. parvum* and *A. cajennense* sialotranscriptomes contained 11 full-length thyropin sequences that were compared with other 15 thyropin sequences from *A. maculatum, A. variegatum*, *Ixodes ricinus*, *Rhipicephalus pulchellu* s, *R. sanguineus* and *Ornithodoros moubata* ticks (Figure 4). The phylogram showed the divergence between hard and soft ticks; the *O. moubata* thyropin sequence (Om-2539) presented low similarity with proteins from hard ticks and clustered as an external group. Two robust super clades (defined by related proteins belonging to clades with > 86% bootstrap support) were composed of three polyspecific clades (with > 72% bootstrap support). Most *Amblyomma* thyropins are more closely related to each other, and some species-specific thyropin sequences were very similar (e.g., Ap-10274, Ap-10275 and Ap-10276 on clade IV), which could represent alleles from the same gene or products of gene duplication. Thyropin sequences found in *A. triste* and *A. cajennense* clustered in clade I and presented higher similarities with *Rhipicephalus* sequences than with proteins in clade IV, which is composed of sequences from all three *Amblyomma* sialotranscriptomes. We observed two consistent subclusters in clade I and IV, formed by *A. triste* and *A. maculatum* sequences, which corroborated the close relationship between these tick species previously described by Estrada-Peña *et al*. [103] through 16S rDNA analysis. The divergence between *Amblyomma* thyropins in clades I and IV is reflected in features such as potential glycosylation sites. A detailed analysis of *A. cajennense* thyropin sequences (performed by the NetOGlyc 4.0 Server) demonstrated that CDS Ac-36285 has one and Ac-103065 has no potential glycosylation sites, while Ac-50867 has 32 potential glycosylation sites (Additional file 3: AF3, column AD). These differences in glycosylation might impact protein functions. Notably, *A. maculatum*, *A. variegatum* and *R. microplus* thyropins possess potential galactosylation sites, suggesting Thyr-1 and mucin domains [43].

**Figure 5:**
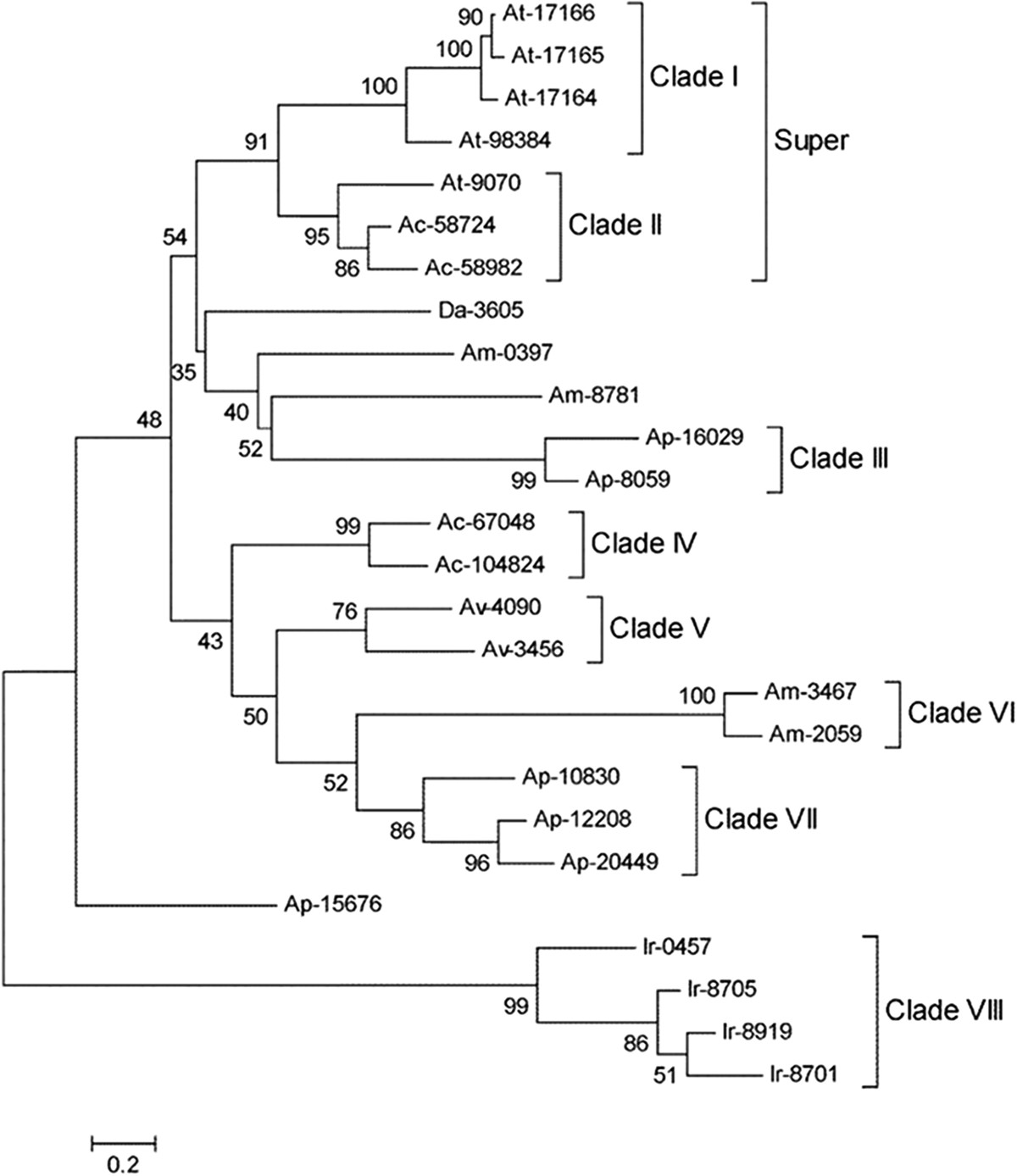
**Phylogenetic analysis of the DAP-36 immunosuppressant proteins from hard ticks.** Phylogram resulting from the alignment of 15 full-length protein sequences from the sialotranscriptomes of *A. triste* (At-17164, At-17166, At-9070, At-98384 and At-17165)*, A. parvum* (Ap-16029, Ap-15676, Ap-10830, Ap-8059, Ap-12208 and Ap-20449) and *A. cajennense* (Ac-67048, Ac-58724, Ac-104824 and Ac-58982) as well as sequences from other hard ticks previously deposited in the NCBI protein database, including *A. maculatum* (Am-3467, Am-2059, Am-8781 and Am-0397)*, A. variegatum* (Av-4090 and Av-3456)*, Ixodes ricinus* (Ir-0457, Ir-8919, Ir-8705 and Ir-8701) and *Dermacentor andersoni* (Da-3605). The database access numbers used in this analysis are listed in Additional file 5: AF5. The neighbor-joining method with 1,000 bootstrap replicates was used for analysis in MEGA 5.05 software. Brackets highlight branches with more than 75% bootstrap support. The bar at bottom indicates 20% amino acid divergence.

Immune modulation is an important requirement for tick feeding because contact with hosts leads to the recognition of tick antigens by immune cells and subsequent activation of both cellular and humoral responses. Suppression of the immune system of hosts facilitates feeding and creates a convenient microenvironment for enhanced pathogen transmission [104]. Ticks develop characteristics to overcome barriers imposed by distinct hosts during their life cycle, and divergent DAP-36 immunosuppressant proteins found in heteroxenous ticks have a general role in immune modulation [105]. This family of proteins has been identified in tick sialotranscriptomes [39, 43, 105] and as immunosuppressants in *Dermacentor andersoni* salivary glands [106]. Fifteen full-length DAP-36 sequences were employed in a phylogenetic analysis with sequences from other hard ticks downloaded from NCBI protein database, including *A. maculatum, A. variegatum, Ixodes ricinus* and *Dermacentor andersoni* (Figure 5). Although the overall analysis showed divergence within the hard ticks, conserved homologues between *A. triste* and *A. cajennense* were shown by the super clade (clade I and II). Notably, clade VIII indicated an earlier deviation of *I. ricinus* DAP-36 proteins from other members of the *Ixodidae* family. As with thyropins, ticks may have developed DAP-36 immunosuppressant proteins through gene duplication, and members of the same clade might have similar functions. Interestingly, CDS Ap-15676 showed little similarity to other *Amblyomma* sequences and was clustered as an external group, suggesting that DAP-36 proteins are divergent within the *Amblyomma* genus. Based on sequences that are >20% divergent at the amino acid level, three putative genes in *A. triste* and *A. parvum* and two putative genes in *A. cajennense* encode members of this family.

## Conclusions

We generated the first detailed transcriptional profile of salivary glands from two important hard ticks from South America, *A. parvum* and *A. triste*, which are reported reservoirs and potential vectors of pathogenic agents such as *Rickettsia*. Additionally, we obtained a larger and more accurate sialotranscriptome of the vector of Brazilian spotted fever, the *A. cajennense* tick. This work extensively supplements the current biological knowledge of the *Amblyomma* genus through the tens of thousands of sequences deposited and freely available for further characterization. Transcripts encoding the protein families important for tick-host-pathogen interactions were common among the libraries and shared similarities with other heteroxenous hard ticks. Thus, these components are good targets for controlling tick infestations and, consequently, decreasing the incidence of tick-borne diseases.

## Authors’ information

All authors were members of a collaborative effort, the GENOPROT: Research Network in Ticks, which was sponsored by the Conselho Nacional de Desenvolvimento Científico e Tecnológico (CNPq) and whose aim was to study the transcriptome and proteome of tick species important for public health and livestock in Brazil. Except for LGG, MMM and HNSM, who have the MSc title, all other authors have the PhD title. SRM is a postdoctoral fellow supported by the São Paulo Research Foundation (FAPESP; fellowships: 2012/15464-0 and 2012/04087-0).

## Electronic supplementary material

Additional file 1: **AF1.** Annotated sialotranscriptome of the *Amblyomma triste tick* (.xlsx), available at http://exon.niaid.nih.gov/transcriptome/sm/AF1-Atris-web.xlsx(DOCX 31 KB)

Additional file 2: **AF2.** Annotated sialotranscriptome of the *Amblyomma parvum tick* (.xlsx), available at http://exon.niaid.nih.gov/transcriptome/sm/AF2-Aparv-web.xlsx(DOCX 32 KB)

Additional file 3: **AF3.** Annotated sialotranscriptome of the *Amblyomma cajennense* ticks, available at http://exon.niaid.nih.gov/transcriptome/sm/AF3-Acaj-web.xlsx(DOCX 31 KB)

Additional file 4: AF4. Functional classification of the LIBEST_USP-RP dataset. (DOCX 26 KB)

Additional file 5: AF5. Accession numbers of the public sequences from other ticks used in phylogenetic analyses. (DOCX 26 KB)

## Abbreviations

aa: Amino acids
APC: Antigen presenting cells
cDNA: Complementary deoxyribonucleic acid
CDS(s): Coding sequence(s)
ESTs: Expressed sequence tags
GRP: Glycine rich proteins
H: Housekeeping
HBP: Histamine binding protein
HCL: Hierarchical clustering
KU: Kunitz domains
LP: *Leishmania*-related proteins
MHC: Major histocompatibility complex
NGP1: *Amblyomma triste* nymphs fed on naïve guinea pigs
NGP2: *Amblyomma triste* nymphs fed on guinea pigs undergoing a second infestation
FD: Female ticks fed on dogs
FD1: *Amblyomma triste* females fed on naïve dogs
FD2: *Amblyomma triste* females fed on dogs undergoing a second infestation
FR: Female ticks fed on rabbits
NSG: Next generation sequencing
nt: Nucleotide
PBS: Phosphate buffered saline
SG(s): Salivary gland(s)
TE: Transposable elements
Thyr-1: Thyroglobulin type-1
TIL: Trypsin inhibitor-like domain
U: Unknown
US: Unknown secreted
V: Viral
S: Secreted.
