# Supplementary material for: The sialotranscriptome of Amblyomma triste, Amblyomma parvum and Amblyomma cajennense ticks, uncovered by 454-based RNA-seq
Source: Parasit Vectors. 2014 Sep 8;7:430. doi: 10.1186/1756-3305-7-430 (PMC4261526; doi:10.1186/1756-3305-7-430)
Supplement: Supplementary file 1 — Additional file 1: AF1. Annotated sialotranscriptome of the Amblyomma triste tick (.xlsx), available at http://exon.niaid.nih.gov/transcriptome/sm/AF1-Atris-web.xlsx(DOCX 31 KB) [file 13071_2014_1606_MOESM1_ESM.docx]

Additional File AF1

Please download the hyperlinked Excel spreadsheet at

[http://exon.niaid.nih.gov/transcriptome/sm/AF1-Atris-web.xlsx](http://exon.niaid.nih.gov/transcriptome/sm/AF1-Atris-web.xlsx)
