# Supplementary material for: The sialotranscriptome of Amblyomma triste, Amblyomma parvum and Amblyomma cajennense ticks, uncovered by 454-based RNA-seq
Source: Parasit Vectors. 2014 Sep 8;7:430. doi: 10.1186/1756-3305-7-430 (PMC4261526; doi:10.1186/1756-3305-7-430)
Supplement: Supplementary file 4 — Additional file 4: AF4. Functional classification of the LIBEST_USP-RP dataset. (DOCX 26 KB) [file 13071_2014_1606_MOESM4_ESM.docx]

**Additional File AF4:** **Functional classification of LIBEST_USP-RP dataset.** A cDNA library from salivary glands of *Amblyomma cajennense* female ticks fed on horses generated by Anatriello *et al*. [1] produced 1,147 Expressed Sequence Tags (ESTs) through Sanger sequencing method. The ESTs were submitted to the bioinformatics workflow developed by Ribeiro *et al*. [2] generating a catalog of 505 contigs containing 997 high quality ESTs, which were functionally annotated based on BLAST results against protein databases, such as NR from NCBI, Gene Ontology, SMART, KOG and PFAM. The ESTs of high quality (except those identical to ribosomal and mitochondrial sequences) are deposited at NCBI dbEST database [GenBank: JZ718403 – JZ718940].

| **Class** | **ESTs** | **%** | **Contigs** | **ESTs/Contigs** |
| --- | --- | --- | --- | --- |
| ***Cytosolic*** |  |  |  |  |
| Protein synthesis | 404 | 40.52 | 50 | 8.08 |
| Energy metabolism | 85 | 8.52 | 58 | 1 |
| Unknown | 180 | 18.05 | 176 | 1.02 |
| Unknown conserved | 57 | 5.71 | 43 | 1.32 |
| Protein modification machinery | 13 | 1.30 | 13 | 1 |
| Transcription machinery | 11 | 1.10 | 9 | 1.22 |
| Nuclear export | 10 | 1.00 | 1 | 10 |
| Cytoskeletal | 9 | 0.90 | 9 | 1 |
| Transporters and storage | 8 | 0.80 | 8 | 1 |
| Signal transduction | 7 | 0.70 | 7 | 1 |
| Oxidative detoxification | 6 | 0.60 | 6 | 1 |
| Detoxification | 6 | 0.60 | 3 | 2 |
| Protein export machinery | 5 | 0.50 | 5 | 1 |
| Proteasome machinery | 4 | 0.40 | 4 | 1 |
| Lipid metabolism | 4 | 0.40 | 4 | 1 |
| Transcription factor | 2 | 0.20 | 2 | 1 |
| Transposable element | 2 | 0.20 | 2 | 1 |
| Carbohydrate metabolism | 2 | 0.20 | 2 | 1 |
| Immunity | 2 | 0.20 | 2 | 1 |
| Nuclear regulation | 1 | 0.10 | 1 | 1 |
| Aminoacids metabolism | 1 | 0.10 | 1 | 1 |
| **Subtotal** | 819 |  | 406 |  |
| ***Secreted*** |  |  |  |  |
| Unknown Secreted | 88 | 8.83 | 64 | 1.375 |
| Basic tail proteins | 35 | 3.51 | 3 | 11.66 |
| Protease inhibitors | 30 | 3.01 | 15 | 2 |
| GYYsuperfamily/cement-like | 16 | 1.60 | 8 | 2 |
| Proteases | 5 | 0.50 | 5 | 1 |
| Extracellular matrix and adhesiom | 2 | 0.20 | 2 | 1 |
| Dap-36 | 1 | 0.10 | 1 | 1 |
| 18.3 kDa subfamily of the Basic tail | 1 | 0.10 | 1 | 1 |
| **Subtotal** | 178 |  | 99 |  |
| **Total** | **997** |  | **505** |  |

**References**

1. Anatriello E, Ferreira BR, Brandão LG, Valenzuela JG, Ribeiro JM, Silva JS, de Miranda Santos IKF: Comparative analysis of transcriptomes of salivary glands from ticks, Amblyomma cajennese and Rhipicephalus sanguineus [abstract]. *Vet Immunol Immunopathol* 2009, 128:223.

2. Ribeiro JM, Alarcon-Chaidez F, Francischetti IM, Mans BJ, Mather TN, Valenzuela JG, Wikel SK: An annotated catalog of salivary gland transcripts from Ixodes scapularis ticks. *Insect Biochem Mol Biol* 2006, 36:111–29.
