# Supplementary material for: The sialotranscriptome of Amblyomma triste, Amblyomma parvum and Amblyomma cajennense ticks, uncovered by 454-based RNA-seq
Source: Parasit Vectors. 2014 Sep 8;7:430. doi: 10.1186/1756-3305-7-430 (PMC4261526; doi:10.1186/1756-3305-7-430)
Supplement: Supplementary file 5 — Additional file 5: AF5. Accession numbers of the public sequences from other ticks used in phylogenetic analyses. (DOCX 26 KB) [file 13071_2014_1606_MOESM5_ESM.docx]

**Additional File AF5: Access numbers of the sequences downloaded from NCBI database, which were used in phylogenetics analyses displayed at Figures 4 (Thyropin) and 5 (DAP-36).**

| **Thyropin sequences** | | | |
| --- | --- | --- | --- |
| ID | Access number | Description | Tick species |
| Am-1991 | gi\|346471991\| | hypothetical protein | *Amblyomma maculatum* |
| Am-8397 | gi\|346468397\| | hypothetical protein | *Amblyomma maculatum* |
| Am-5097 | gi\|346465097\| | hypothetical protein | *Amblyomma maculatum* |
| Am-4869 | gi\|346464869\| | hypothetical protein | *Amblyomma maculatum* |
| Av-3222 | gi\|325303222\| | TPA_inf: putative thyropin precursor | *Amblyomma variegatum* |
| Ir-1199 | gi\|442751199\| | Putative two thyropin domains protein | *Ixodes ricinus* |
| Ir-0875 | gi\|442750875\| | Putative two thyropin domains protein | *Ixodes ricinus* |
| Ir-1197 | gi\|442751197\| | Putative two thyropin domains protein | *Ixodes ricinus* |
| Ir-1195 | gi\|442751195\| | Putative two thyropin domains protein | *Ixodes ricinus* |
| Om-2539 | gi\|41352539\| | putative thyropin precursor | *Ornithodoros moubata* |
| Rp-8045 | gi\|427798045\| | Putative tick salivary thyropin, partial | *Rhipicephalus pulchellus* |
| Rp-0427 | gi\|427790427\| | Putative tick salivary thyropin | *Rhipicephalus pulchellus* |
| Rp-8209 | gi\|427778209\| | Putative tick salivary thyropin | *Rhipicephalus pulchellus* |
| Rp-0373 | gi\|427790373\| | Putative tick salivary thyropin | *Rhipicephalus pulchellus* |
| Rs-8564 | gi\|260908564\| | Putative thyropin precursor | *Rhipicephalus sanguineus* |

| **DAP-36 immunosuppressant family** | | | |
| --- | --- | --- | --- |
| ID | Access number | Description | Tick species |
| Ir-0457 | gi\|442760457\| | Putative dap-36 protein member | *Ixodes ricinus* |
| Ir-8919 | gi\|442758919\| | Putative dap-36 protein member | *Ixodes ricinus* |
| Ir-8705 | gi\|442758705\| | Putative dap-36 protein member | *Ixodes ricinus* |
| Ir-8701 | gi\|442758701\| | Putative dap-36 protein member | *Ixodes ricinus* |
| Am-3467 | gi\|346473467\| | hypothetical protein | *Amblyomma maculatum* |
| Am-2059 | gi\|346472059 | hypothetical protein | *Amblyomma maculatum* |
| Am-8781 | gi\|346468781\| | hypothetical protein | *Amblyomma maculatum* |
| Am-0397 | gi\|346470397\| | hypothetical protein | *Amblyomma maculatum* |
| Da-3605 | gi\|6103605\| | immunosuppressant protein p36 | *Dermacentor andersoni* |
| Av-4090 | gi\|325304090\| | TPA_inf: dermacentor immunosuppressant protein p36-like protein | *Amblyomma variegatum* |
| Av-3456 | gi\|325303456\| | TPA_inf: dermacentor immunosuppressant protein p36-like protein | *Amblyomma variegatum* |
